# Monitoring the Effect of Weed Encroachment on Cattle Behavior in Grazing Systems Using GPS Tracking Collars

**DOI:** 10.3390/ani13213353

**Published:** 2023-10-28

**Authors:** Igor L. Bretas, Jose C. B. Dubeux, Priscila J. R. Cruz, Luana M. D. Queiroz, Martin Ruiz-Moreno, Colt Knight, Scott Flynn, Sam Ingram, Jose D. Pereira Neto, Kenneth T. Oduor, Daniele R. S. Loures, Sabina F. Novo, Kevin R. Trumpp, Javier P. Acuña, Marilia A. Bernardini

**Affiliations:** 1 North Florida Research and Education Center, University of Florida, Marianna, FL 32446, USAldantasqueiroz@ufl.edu (L.M.D.Q.); ruizm001@ufl.edu (M.R.-M.); kenneth.oduor@ufl.edu (K.T.O.); novosabina@gmail.com (S.F.N.); ktrumpp@ufl.edu (K.R.T.); portuguez.javier@ufl.edu (J.P.A.); m.araujobernardi@ufl.edu (M.A.B.); 2 Range Cattle Research and Education Center, University of Florida, Ona, FL 33865, USA; prodriguesdacruz@ufl.edu; 3 University of Maine Cooperative Extension, Orono, ME 04469, USA; colt.knight@maine.edu; 4 Corteva Agriscience, Lee’s Summit, MO 64015, USA; scott.flynn@corteva.com (S.F.); sam.ingram@corteva.com (S.I.); 5 Department of Animal Science, Auburn University, Auburn, AL 36849, USA; jdp0130@auburn.edu; 6 Departament of Animal Science, Universidade Federal do Recôncavo da Bahia, Cruz das Almas 44430-622, BA, Brazil; drloures@ufrb.edu.br

**Keywords:** animal activity, geographic information system, global navigation satellite system, grasslands, livestock monitoring, weed management

## Abstract

**Simple Summary:**

Pasture weed encroachment is a major challenge in livestock production based on grazing systems. *Amaranthus spinosus* L. is an annual weed species with high invasive potential worldwide, and it can affect animal behavior and well-being due to the presence of sharp spines. We used GPS tracking collars to monitor cattle activity and spatial distribution in a grazing system with different levels of weed encroachment. Animals in weed-infested paddocks had reduced resting time and increased grazing time, distance traveled, and rate of travel compared to animals in weed-free paddocks. The spatial distribution of the cattle was greater in weed-free paddocks than in weed-strips or weed-infested areas. Pasture weed encroachment affects cattle behavior and their spatial distribution across the pasture. Increasing animal activity can result in lower animal performance due to greater energy expenditure and impacts on animal welfare. Understanding the impact of pasture weed encroachment on animal behavior is especially important for increasing livestock productivity and sustainability while maintaining animal welfare.

**Abstract:**

Weed encroachment on grasslands can negatively affect herbage allowance and animal behavior, impacting livestock production. We used low-cost GPS collars fitted to twenty-four Angus crossbred steers to evaluate the effects of different levels of weed encroachment on animal activities and spatial distribution. The experiment was established with a randomized complete block design, with three treatments and four blocks. The treatments were paddocks free of weeds (weed-free), paddocks with weeds established in alternated strips (weed-strips), and paddocks with weeds spread throughout the entire area (weed-infested). Animals in weed-infested paddocks had reduced resting time and increased grazing time, distance traveled, and rate of travel (*p* < 0.05) compared to animals in weed-free paddocks. The spatial distribution of the animals was consistently greater in weed-free paddocks than in weed-strips or weed-infested areas. The effects of weed encroachment on animal activities were minimized after weed senescence at the end of the growing season. Pasture weed encroachment affected cattle behavior and their spatial distribution across the pasture, potentially impacting animal welfare. Further long-term studies are encouraged to evaluate the impacts of weed encroachment on animal performance and to quantify the effects of behavioral changes on animal energy balance.

## 1. Introduction

It is estimated that agricultural production needs to increase by approximately 50% by 2050 to meet the world food demand due to global population growth and rising income [1]. Livestock products such as milk and meat are among the most consumed foods worldwide [2]. Grasslands account for around 70% of the global agricultural land, representing a crucial feed source to increase livestock production [3]. However, weeds are a major limiting factor for world agricultural livestock production [4,5].

Using herbicides is the most common and effective method of weed control [6]. However, the environmental impacts and costs of chemical applications in grasslands make weed management often overlooked in grasslands. Weed encroachment on grasslands negatively affects livestock production by reducing herbage allowance and forage nutritive value due to competition for limited resources such as nutrients, water, and light with desirable plants [4,7]. In grazing systems, weeds can also affect animal welfare and behavior because of toxic components and spine injuries [2,8]. 

The genus *Amaranthus* accounts for approximately 70 species across the globe [9]. Spiny pigweed (*Amaranthus spinosus* L.) is an annual small-seeded, broadleaf weed species with extended germination, fast growth, high seed production, and high invasive potential. It is widely found in grasslands [10]. Furthermore, spiny pigweed presents sharp spines at the axils of the leaves and branches. Spines can impact animal behavior and well-being in grazing systems [11]. Typically, spiny pigweed is not palatable, and grazing animals usually avoid grazing spiny-pigweed-infested areas because of the spines [12,13]. Spiny pigweed has been also associated with nitrate accumulation in livestock [12], although toxicity effects in cattle are not well documented.

In addition to the demand for increasing livestock production efficiency, there is growing global concern regarding animal welfare in livestock production systems. In Florida (USA), spiny pigweed usually encroaches on paddocks spontaneously during the warm season (spring–summer). More investigation into the impact of weed encroachment on animal behavior is required in order to better explain the animal performance responses. Several studies have demonstrated the effects of weed encroachment on forage responses [2,7,14,15], but studies evaluating the effect of weeds on animal responses are scarce. Regarding livestock responses, most scientific studies focus on performance without evaluating changes in animal behavior or well-being, which can be a reason for changes in animal performance. The difficulty of monitoring animal behavior in grazing systems can explain the lack of studies. Traditional methods applied to animal behavior monitoring in grazing systems are based on field observations by a trained observer, which is laborious and time-consuming, and sometimes can influence normal animal behavior [16,17]. 

Advances in precision livestock farming technologies allow the use of global positioning systems (GPS) to monitor animal behavior on grazing systems remotely in near real-time. Using low-cost GPS and geographic information systems (GIS) enables the monitoring of the spatial variability of animal distribution over time in grazing areas [18]. Understanding the site use preferences of grazing animals is crucial for developing a more sustainable livestock grazing system [19]. GPS collars can also be used to estimate grazing animals’ activities, such as grazing time, resting time, and traveling time, based on previously calibrated patterns of animals’ movements [20,21]. 

Freedom to express normal behavior is one of the five defining principles of animal welfare [22]. Therefore, monitoring daily variations in animal activities helps to characterize the typical pattern of animal behavior and detect possible deviations [23] which may indicate the animals’ welfare status in grazing systems. The applicability of low-cost GPS and GIS data to monitor animal distribution and activity simultaneously can be a cost-effective strategy to reduce the necessity of tri-axis accelerometers, which are typically used for activity monitoring.

Most studies using GPS collars to track livestock behavior have been set to collect GPS signals every 5 to 15 min during short periods [19,24], preventing the tracking of smooth changes, such as sinuosity of movements, in animal behavior throughout the day, as well as their variability over time. Furthermore, as far as we are aware, there are no studies evaluating the impacts of weed encroachment on bermudagrass (*Cynodon dactylon* (L.) Pers.) pastures on animal behavior. Therefore, this study aimed to evaluate the effect of pasture weed encroachment on animal behavior through GPS collars, which registered signals every second for a lengthy period of time. We hypothesized that animals would avoid weed-infested areas in the paddock and increase their daily activity as weed encroachment increased.

## 2. Materials and Methods

### 2.1. Site Description and Experimental Design

**Figure 1:**
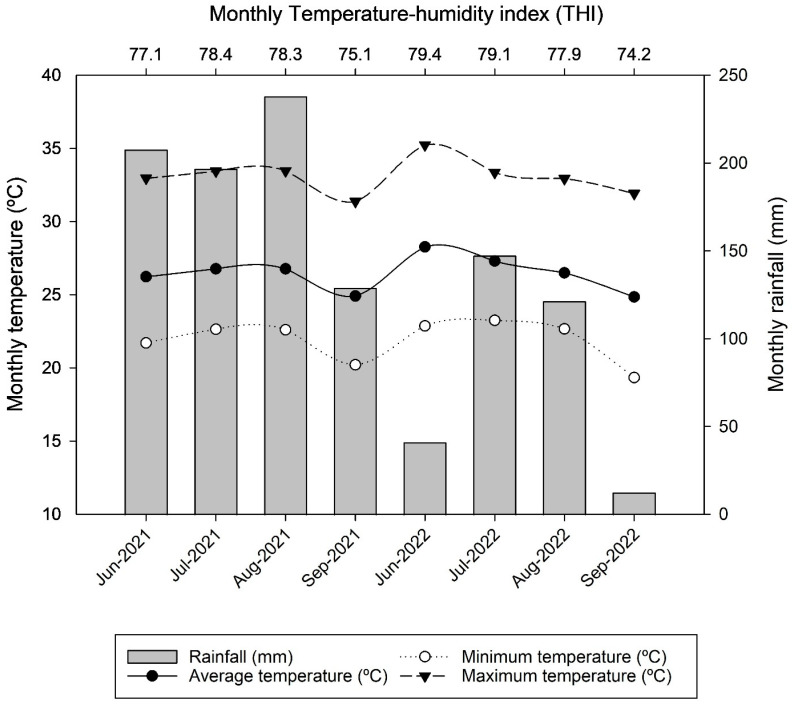
Rainfall, temperature (average, minimum, and maximum), and temperature–humidity index (THI) during the experimental periods in 2021 and 2022. Data were obtained from the Florida Automated Weather Network (FAWN).

This study was carried out at the University of Florida, Institute of Food and Agricultural Sciences (UF/IFAS), North Florida Research and Education Center, Marianna, FL, USA, during the summers (June to September) of 2021 and 2022. Twelve paddocks (30°51′47.96″ N; 85°11′4.78″ W) of common bermudagrass, approximately 1.3 ha each in area, were established (*Cynodon dactylon* (L.) Pers.). Climatic data for the experimental period were collected at the Florida Automated Weather Network (FAWN). The monthly accumulated rainfall, temperature (average, minimum, and maximum), and temperature–humidity index (THI) for both experimental years (2021 and 2022) are reported in Figure 1. The THI was calculated using the dry bulb temperature (Tdb, °C), and relative humidity (%; RH) according to the equation described by [25].

(1) $$THI=\left(1.8\times Tdb+32\right)-[\left(0.55-0.0055\times RH\right)\times \left(1.8\times Tdb-26.8\right)]$$

**Figure 2:**
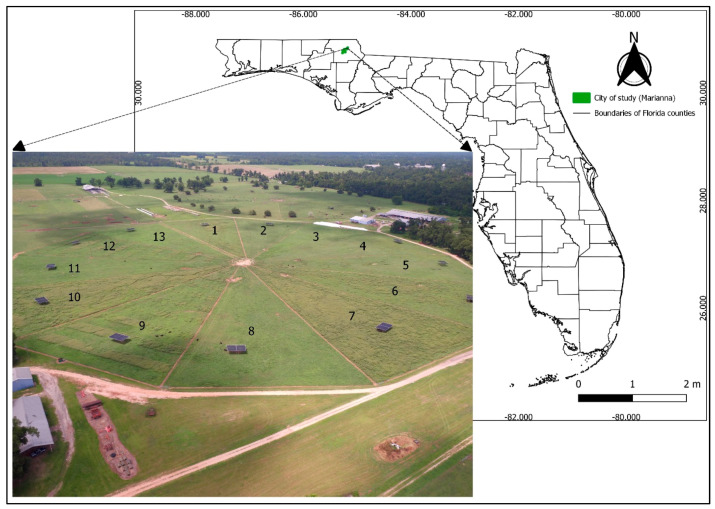
Map of Florida and drone image highlighting the experimental site. Paddocks 2, 4, 8, and 11 were weed-free; paddocks 5, 9, 12, and 13 were established with alternated weed-strips; and paddocks 1, 6, 7, and 10 were weed-infested. Paddock 3 was not used.

The experiment was established with a randomized complete block design, with three treatments and four blocks, totaling twelve experimental units (paddocks). The treatments were paddocks free of weeds (weed-free), paddocks with weeds established in alternated strips (weed-strip), and paddocks with weeds spread throughout the entire area (weed-infested; Figure 2).

To establish the treatments, spiny pigweed seeds were drilled (1.1 kg ha^−1^) in the uncontrolled (weed-infested) and alternated strip paddocks in May 2020. All of the seeds were collected at the research station during the summer of 2020. In 2021, we sprayed Corteva Agriscience^TM^ DuraCor^TM^ herbicide (Aminopyralid 8.95% + Florpyrauxifen-benzyl 0.76%; 1.5 L/ha) and used 2.5 mL/L of non-ionic surfactant (Alkyl Aryl Polyoxyethylene Glycols, free fatty acids, and Isopropanol 80%) to set the treatments. The herbicide was applied only for the weed-free and weed-strip treatments. Thus, for the “weed-free” treatment, the whole area was sprayed with the herbicide at the beginning of the experiment (early June). Similarly, the spiny pigweed strips were established using the same herbicide, with the strips’ coordinates previously inserted into the system of the sprayer tractor. The “weed-infested” treatment paddocks were not sprayed with any herbicide. The treatments were designed to create different levels of weed encroachment in the pasture. The same spraying protocol was repeated in early June of 2022 for the second year of evaluations.

### 2.2. Forage and Animal Evaluations

The Institutional Animal Care and Use Committee (IACUC) approved the experimental protocols (IACUC202200000506). Two crossbred Angus steers (*Bos* spp.) per paddock were considered testers and remained on the respective treatments throughout each cycle of study (summer 2021/2022). The initial mean BWs of the tester steers (*n* = 24) were 358 ± 22 and 387 ± 29 kg for 2021 and 2022, respectively. All of the paddocks were continuously stocked under variable stocking rates using “put-and-take” animals to adjust the herbage allowance. The target herbage allowance was 2 kg DM of bermudagrass herbage mass per kg BW for all treatments. The put-and-take animals were extra animals added to or removed from each paddock every cycle (21 d), according to the measured bermudagrass herbage mass (HM), to maintain the same herbage allowance for all the treatments during the entire experimental period. We used a high herbage allowance threshold to guarantee enough fodder for the animals and avoid possible issues with toxicity in weed-infested paddocks. The bermudagrass HM was quantified every 21 d using double sampling [26,27] and destructive method techniques. For the weed-free treatment, the double sampling method was utilized, for which thirty disk heights were taken at random sites within each paddock. The bermudagrass HM at each double sampling site (4 per paddock) was clipped at ground level using a known-area frame (0.25 m^2^) and then dried at 55 °C for 72 h to determine the actual herbage mass. A calibration equation for the disk was developed every 21 d, with the measured HM regressed on the disk settling height. For the weed-infested treatment, thirty bermudagrass disk heights were collected, and botanical composition was measured after clipping eight representative points at ground level in each paddock. Both species were dried to determine the actual herbage mass of each. The herbage mass of bermudagrass in weed-infested areas was then determined by calculating the average of both methods (double sampling and destructive method). For the weed-strip treatment, sixty bermudagrass disk heights were measured. Thirty disk measurements were taken in the striped area encroached upon by spiny pigweed, and the other thirty were taken in the bermudagrass strip. Botanical composition was measured after clipping four representative points at ground level in the spiny pigweed strips only. The material was then dried to assess the actual mass of the herbage from both species in the spiny pigweed strip. Similarly to the weed-infested treatment, the herbage mass of bermudagrass was determined by calculating the average of both methods (the double sampling and destructive methods). This approach of averaging both methods was adopted in order to consider the bermudagrass growing beneath the spiny pigweed cluster. To determine the spiny pigweed mass in the weed-strip treatment paddocks, the herbage mass obtained by the destructive method was adjusted for the spiny pigweed area in the paddock (~48%; [28]). The animals were weighed every 21 d after 16 h of withdrawal from feed and water to estimate the average daily gain (ADG) and adjust the stocking rate. Water, shade, and a mineral mixture were available ad libitum. The herbage allowance was also measured every 21 days using the method described by [29].

### 2.3. GPS Collars and Data Collection 

Two tester steers from each paddock were fitted with GPS collars, for a total of twenty-four tester animals distributed among the treatments (2 animals × 3 treatments × 4 blocks). The GPS collars used in this study were constructed as described by [30] using the Mobile Action igot-U 120 travel and sports loggers (Mobile Action Technology, New Taipei City, Taiwan), adapted using a rechargeable battery with a greater capacity and traditional leather working tools. According to Morris [31], the estimated average location error for this device was <10 m, varying according to location and fix frequency, with almost 100% of the successful fix rate. 

The devices were set to record positions (latitude/longitude) every second for twenty-one days. This period was chosen according to the device’s battery lifespan and the schedule of forage and animal evaluations during the trial. The animals were monitored for four sequential periods of 21 d in 2021 and five periods (21 d) in 2022, totaling 84 days of monitoring in 2021 and 105 days in 2022. The evaluation periods are described in Table 1. We opted to extend the evaluation period in the second year of study to track possible changes in animal behavior after weed senescence and a reduction in weed encroachment. After each 21-day period, the animals were led to the pen, and the GPS collars were removed, recharged for 24 h, and re-fitted the following day. For data filtering, we plotted the GPS points using QGIS^®^ (v. 3.22.14) software and created shapefiles of each paddock boundary for the study area. The paddock boundaries were defined using a georeferenced Google Earth image and fence posts as ground control points. All of the GPS coordinates registered out of the respective paddock boundary, including data registered along the pathway from the pen to the paddock, were filtered and excluded. We did not filter inaccurate locations inside the respective paddocks, since the inaccuracy was the same for all treatments. Filtering inaccurate locations could result in the loss of many locations, with a low error in return for a slight improvement in location precision [31].

### 2.4. Data Processing and Analysis

#### 2.4.1. Animal Activity

To calculate the distance between two sequential points and to handle data on GIS, latitude and longitude coordinates were converted to the Universal Transverse Mercator (UTM) coordinate system using the GSTAT, SP, and RGDAL packages in R Studio^®^ (R version 4.3.0). The distance between two sequential positions was calculated using the Pythagorean theorem, then summed for 24 h periods to estimate the distance traveled per day by each animal (m/day). The rate of travel (m/min) was calculated using the ratio between the traveled distance and time spent to change the location point. The daily distance traveled and rate of travel were calculated using R Studio^®^ (Posit software, R version 4.3.0) programming. The data from each paddock were averaged across the two animals (testers).

To estimate the daily patterns of animal activity, including grazing time, resting time, and traveling time, we used the rate of travel pattern validated by Augustine [32], adapted by Nyamuryekung’e [21], and described by McIntosh [20] for steers in grazing systems. The referenced studies suggest classifying the animal activity according to speed, assuming a speed lower than 2.34 m/min as resting, a speed between 2.34 m/min and 25 m/min as grazing, and a speed greater than 25 m/min as traveling. Therefore, all stationary activities were classified as resting, including rumination. We used R Studio^®^ (Posit Team, 2023) programming to calculate the percentage of time spent on each activity daily. The data were averaged across the two animals from each paddock.

#### 2.4.2. Livestock Spatial Distribution

**Figure 3:**
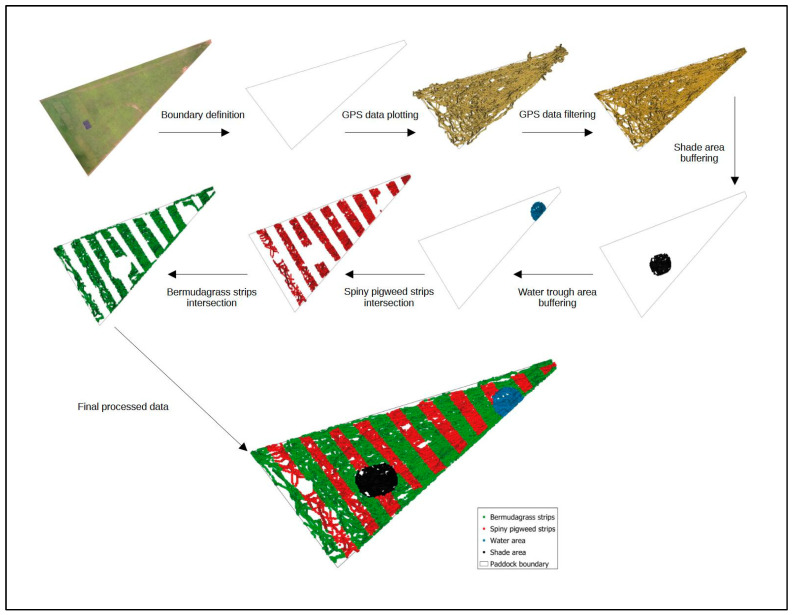
Flowchart of the global positioning system (GPS) data processing using geographic information system (GIS) mapping.

To estimate the daily time spent (%) by the animals in areas of water and shade, as well as the time spent in each strip (bermudagrass × spiny pigweed) in the weed-strip treatment paddocks, we used the vector tools of the referred GIS software (QGIS^®^ v. 3.22.14) to generate an additional shapefile for each spot of interest (i.e., shade, water, bermudagrass strips, and spiny pigweed strips). The geographic coordinates of each corner of the artificial shades, water troughs, and weed strips had previously been taken by a conventional GPS using a smartphone (Coordinates-GPS Formatter app V. 7.8.0). To minimize the inaccuracy of the smartphone GPS, we rasterized the vector points and used a georeferenced Google Earth image and the “georeferencer” tool in QGIS^®^ software to adjust the shade and water trough areas. To consider the shade projection, the area around the water troughs, and the remaining inaccuracy of the smartphone–GPS system, we generated a new shapefile for shade and water troughs with a round buffer of 10 m from the original coordinate using the geoprocessing “buffer” tool of the software QGIS^®^. To estimate the percentage of daily time spent near shade or water, we used the spatial analyst tools in the same mapping program to calculate the percentage of GPS points inside of each buffered area (shade or water) through the intersection between the filtered GPS points and the respective area of interest. For weed-strip treatments, the same approach was adopted to estimate the percentage of time spent by grazing animals in the bermudagrass strips or spiny pigweed strips. Data were averaged across five evaluation periods and two years of study for each treatment. Figure 3 represents a flowchart of the data processing in the GIS software (QGIS^®^ v. 3.22.14).

To track the animal spatial distribution and identify the animals’ site preferences, we generated a heatmap based on the filtered GPS data using the Kernel density method of the software QGIS^®^. This interpolation method created a density map of an input vector layer (GPS points) using the Kernel density estimation. The density was calculated based on the number of points in a location, with larger numbers of clustered points resulting in larger values. Because of the high requirement for computing resources and the hard work necessary to process the big data within a reasonable amount of time, we randomly selected one paddock from each treatment in each study year to generate heatmaps and characterize the pattern of animal distribution. To estimate the livestock site preferences, we calculated the Landscape Preference Index (LPI) using the ratio of the proportion of time spent in the area of interest to the proportion of the area of interest compared to the entire available area, as described in Handcock [33].

### 2.5. Statistical Analysis

We computed daily means for each collared steer and then calculated the average for each paddock (two animals) in each treatment. The statistical model used for the analyses was:

Yijk = μ + τi +βi + tk + (τ × t) ik + εijk

where Yijk is the observation ijk; μ is the overall mean; τi is the effect of treatment i; βi is the effect of block; tk is the effect of period k; (τ × t) ik is the effect of interaction between treatment i and period k; and εijk is the random error. Data analysis was performed using PROC MIXED from SAS version 9.1 (SAS Institute, Cary, NC, USA). An analysis of variance was carried out, considering the year (2021/2022) as a random effect. Differences between treatments were considered significant at *p* < 0.05.

## 3. Results

### 3.1. Forage and Animal Responses

There was no interaction between treatments and evaluation periods for herbage or animal responses (*p* > 0.05). There were no differences in bermudagrass HM, ADG, stocking rate, or herbage allowance among treatments (*p* > 0.05; Table 2). The spiny pigweed HM was greater in weed-infested paddocks compared to weed-strips (*p* < 0.05; Table 2). 

**Figure 4:**
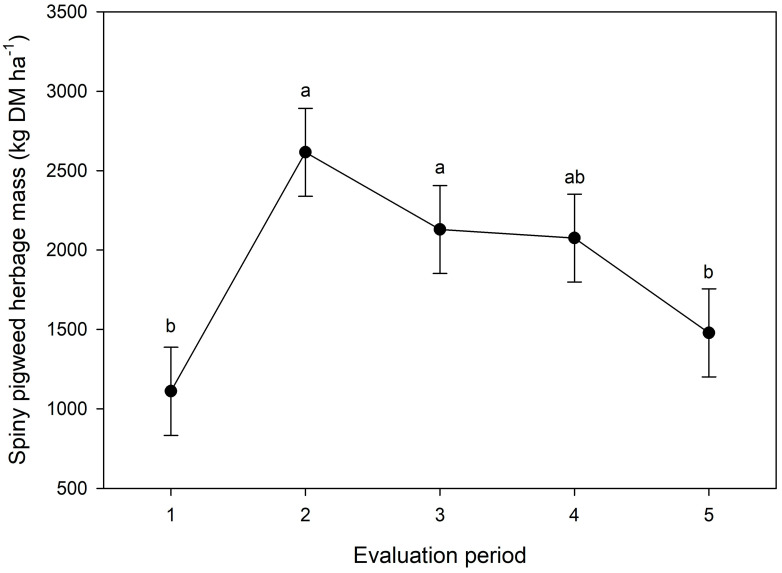
Spiny pigweed herbage mass in different periods of evaluation, averaging the weed-infested and weed-strip areas. Means presented with different letters are significantly different (*p* < 0.05).

The spiny pigweed HM was also affected by the evaluation period (*p* < 0.05), with a peak of HM in the second evaluation period and lower HM observed for the first and last periods of evaluation (Figure 4). 

### 3.2. Animal Activity

The average daily distance traveled varied from 4482 to 5657 m over the two evaluation cycles (summer 2021 and 2022). There was a significant effect of pasture weed encroachment on the daily distance traveled by the animals (*p* < 0.05), with animals in weed-infested paddocks increasing their daily walking by approximately 26% compared to animals grazing in weed-free paddocks (Table 3). Similarly, animals grazing in weed-infested paddocks presented a greater average daily travel rate than the other treatments (Table 3).

**Figure 5:**
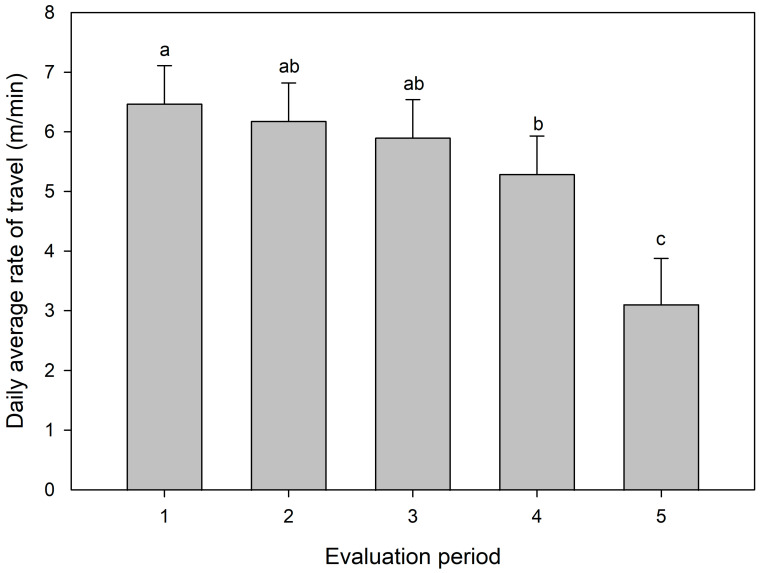
Daily average grazing rate of travel for the animals throughout five evaluation periods. Bars with different letters are significantly different (*p* < 0.05).

There was no effect of the evaluation period and no interaction between the treatment and evaluation periods on the distance the animals traveled throughout the study (*p* > 0.05). However, the evaluation period significantly affected the travel rate of grazing steers (*p* < 0.05; Figure 5). Overall, cattle traveled at greater rates during the first three evaluation periods, tending to reduce the travel rate in the last two periods (Figure 5). The lowest rate of travel was in the last evaluation period (*p* < 0.05; Figure 5).

Besides the traveled distance and rate of travel, weed encroachment also affected the other daily activities of grazing animals. Animals in weed-infested paddocks showed significantly reduced resting time compared to those in weed-free paddocks (*p* < 0.05), while increasing the grazing time (*p* < 0.05) compared either with weed-strips or weed-free paddocks (Table 4). There was no effect of weed encroachment levels on daily traveling time despite the tendency of grazing animals to increase their daily traveling time in weed-infested paddocks (Table 4).

**Figure 6:**
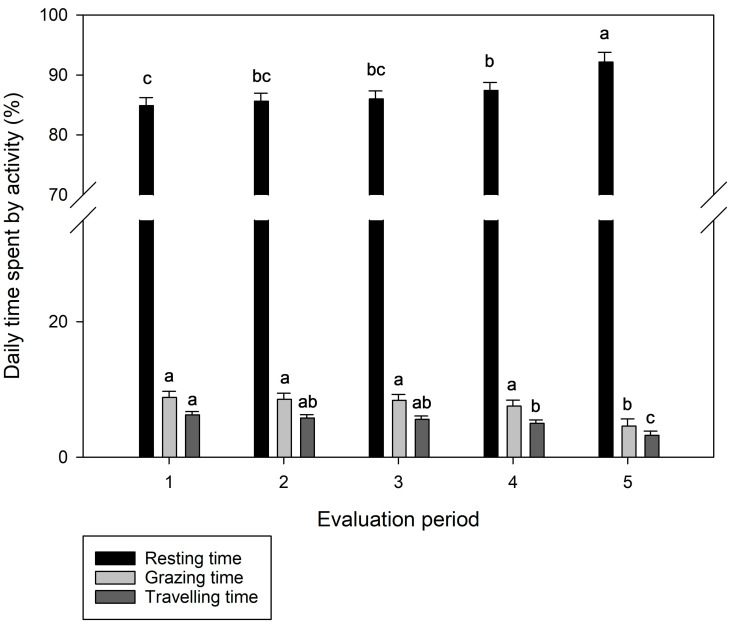
Daily time spent by grazing animals engaging in different activities throughout five evaluation periods. a–c LSMeans followed by the same letter did not differ among treatments at *p* < 0.05. Error bars represent ± standard errors of the means.

There was a significant effect of the evaluation period on resting time, grazing time, and traveling time (*p* < 0.05), with no interaction between the level of weed encroachment and the evaluation period (*p* > 0.05). Overall, animals presented longer resting times in the last evaluation period compared to the other four (Figure 6). Contrarily, animals presented greater grazing times during the first four evaluation periods, reducing their grazing times in the last evaluation period. A similar pattern was observed for traveling time, which was reduced during the last period of evaluation (Figure 6).

### 3.3. Livestock Spatial Distribution

Overall, on average, the animals remained in shady areas for 5 h (21% of the day) and in water trough areas for 2.6 h (11%). There were no significant differences (*p* > 0.05) in the time spent in shady or water trough areas by grazing animals according to the different levels of pasture weed encroachment. However, there was a tendency to spend less time by water trough areas in weed-infested paddocks (Table 5).

Regarding the animal behavior in paddocks with spiny pigweed established in alternated strips with bermudagrass, we noticed that the animals stayed for longer periods of time in bermudagrass strips than in spiny pigweed strips (*p* < 0.05; Table 5). The animals remained in bermudagrass strips for, on average, 8.9 h (37% of the day), and in spiny pigweed strips for 7 h (29% of the day). When the time spent by each area (i.e., water, shade, or strips) was adjusted to the occupied proportion of the paddock area (LPI), areas near shade and water presented greater indices. Similarly, bermudagrass strips had a greater index compared to spiny pigweed strips (Table 5).

**Figure 7:**
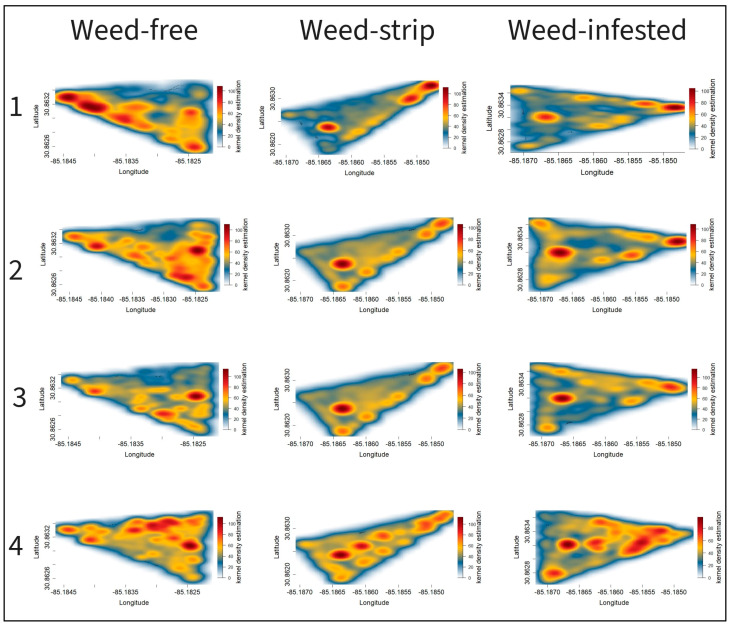
Heatmaps of grazing animals’ distribution based on kernel density estimation for different levels of pasture weed encroachment during four evaluation periods in 2021. The left number represents the evaluation periods (1–4), and areas in dark red represent hotspots of animal distribution.

**Figure 8:**
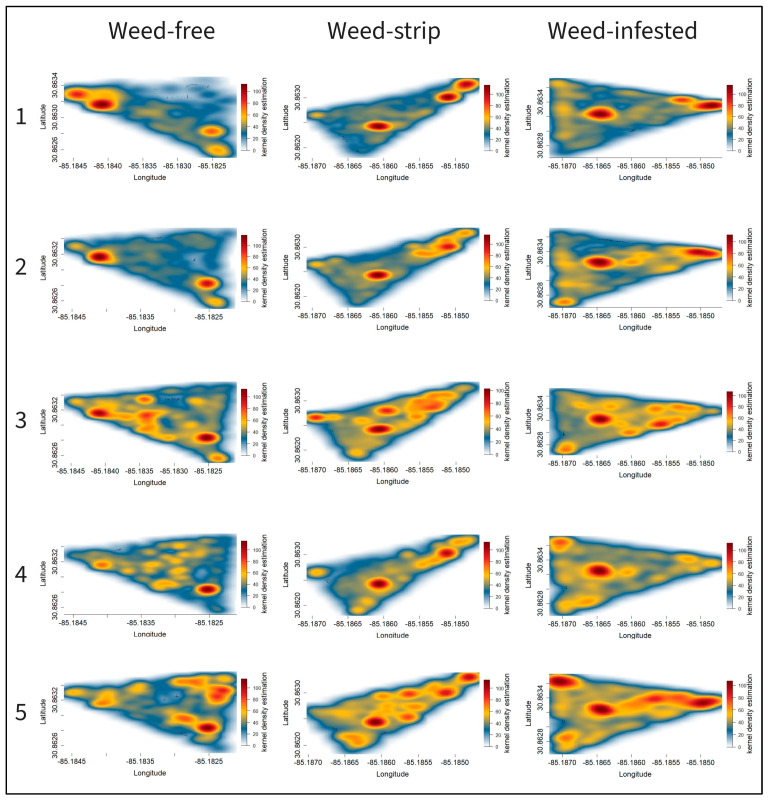
Heatmaps of grazing animals’ distribution based on kernel density estimation for different levels of pasture weed encroachment during four evaluation periods in 2022. The left number represents the evaluation periods (1–5), and areas in dark red represent hotspots of animal distribution.

Figure 7 and Figure 8 represent the heatmaps based on kernel density estimation for different treatments in 2021 and 2022, respectively. Regardless of the treatment, shady areas, water trough areas, and areas close to the gates were the hotspots for animal distribution throughout the study.

The heatmap from the two complete evaluation cycles (2021/2022) demonstrated the same pattern of animal distribution according to the treatment and period of evaluation. During the first three periods of evaluation, the animals in paddocks encroached by weeds (weed-strips or weed-infested) were concentrated in the hotspots mentioned above and moved mainly through the edges of the paddock, with this behavior being more evident for weed-infested paddocks and in 2021. The animals spread out across the paddock area from the third evaluation period onwards, progressively occupying larger paddock areas (Figure 7 and Figure 8). The greater spatial distribution of animals was greater in the last period of evaluation for both years. Notably, animals in weed-free paddocks presented a more homogeneous spatial distribution over the five study periods in both years.

## 4. Discussion

### 4.1. Forage and Animal Responses

The absence of effects of weed encroachment on bermudagrass herbage allowance can be explained by the bermudagrass growth beneath the spiny pigweed clusters, which was associated with the low grazing intensity used in this trial to avoid animal injuries or toxicity issues with the weeds. The aim of this study was to evaluate the impacts of pasture weed encroachment on animal behavior, assuming that behavioral changes could impact long-term animal performance. We set all the treatments with the same herbage allowance to control the effect of herbage allowance on animal behavior. Thus, the same animal performance observed among treatments in this study was also expected due to the same herbage allowance and the brief period of evaluation for inferences in terms of animal performance. Our findings suggest that the animals were able to adapt to weed-infested areas by changing their activity patterns, possibly to intensify their forage searching. It is possible that the period of study was too short to detect the impact of increasing animal activity on energy expenditure and, consequently, animal performance, although we did not evaluate the animal energy balance. Also, it is possible that reducing the herbage allowance by increasing the stocking rate could result in greater differences among the treatments, since the animals would face more challenges in terms of browsing forage, and the competition would increase. Additionally, we did not offer any supplement besides mineral mixture to the animals, which prevented greater ADG that could provide evidence for differences among the treatments. 

### 4.2. Animal Activity

The average daily distance traveled which was estimated in this study (~5 km) is similar to the one observed by Johnson [34]. It was discovered by tracking grazing cattle in every-second intervals using GPS collars. Knight [31] also used the same device to track grazing cattle and estimated a daily traveled distance of about 6 km. On the other hand, McIntosh [20] found a greater average traveled distance by grazing animals during the summer (9.3 km). However, that study was conducted on rangeland of a larger area (~3200 ha), which might have increased the forage-searching activity of the animals. McGavin [35] demonstrated that paddock size affected distances traveled by cattle, with distances increasing as the paddock size increased. Overall, paddock size, distance to water, topography, forage allowance, animal nutritional requirement, temperature, humidity, rainfall, and other factors intrinsic to each site can also impact the daily distance traveled by grazing animals, which makes it difficult to compare the traveled distances estimated by different studies. Moreover, the GPS sampling frequency also affects the estimated daily distance traveled. Refs. [35,36] evaluated the effects of GPS collar sampling frequency on the distance traveled by cattle per day, and demonstrated that shorter sampling intervals overestimated the daily distance traveled. Conversely, extending the GPS sampling intervals was shown to underestimate daily traveled distances [35]. The overestimation of traveled distances can be explained by accumulated measurement errors, while underestimation can be explained by interpolation errors. Measurement error refers to the difference between the recorded GPS position and the true position, while interpolation error refers to the sinuosity movements lost by assuming linear movements between points [37]. Thus, our study likely minimized the interpolation error, but incorporated some measurement errors. Indeed, there is a trade-off in determining the best GPS sampling frequency for cattle monitoring [37]. McGavin [35] suggested GPS sampling intervals from every 5 to 10 s as the optimum for minimizing both errors; however, the optimum sample rate can vary according to device accuracy, animal species, topography, and objective of the monitoring, thus requiring specific evaluations. Our study was focused on the effects of weed encroachment level on cattle behavior; therefore, regardless of the measurement errors added to our estimated traveled distances, the same errors were incorporated into all treatments, allowing for a comparison among them.

The greater daily travel distances observed for grazing animals in weed-infested areas compared to areas with lower levels of weed encroachment or weed-free areas may be justified by the challenge animals face finding fodder in weed-infested areas. According to Owen-Smith [38], extended traveling can be associated with forage seeking. Animals will likely travel greater distances in weed-infested areas to meet their nutrient requirements, possibly due to the difficulty of accessing forage. Greater travel distances can potentially affect animal performance due to greater energy expenditure [39]. Most animal energy expenditure above the metabolic rate is due to locomotion [40]. In addition to energy expenditure monitoring, changes in the patterns of daily traveled distance can be used as an indicator of animal welfare status or for estrus and disease detection [41,42,43,44]. 

Grazing animals in weed-infested paddocks also presented a greater average rate of travel. Animals traveled longer distances and moved faster through different grazing stations in weed-infested paddocks as a possible adaptation in order to maintain the same dry matter intake in a similar period. Spine pigweed clusters might have restricted the forage access in some spots, mainly access to the forage growing beneath the weed cluster, due to the spines. This explanation is supported by the effect of the evaluation period on the rate of travel. The travel rate significantly decreased in the last period of evaluation (late summer), when the spiny pigweed was becoming senescent and had a lower HM. The pattern of the rate of travel from the second to the last evaluation periods followed a similar pattern to spiny pigweed HM. These findings suggest that the animals remained for longer in the same grazing station because access to the forage was growing easier. Accordingly, the greater resting time and reduced grazing time in the last period of evaluation also demonstrate the effect of spiny pigweed HM on daily animal activities. 

The greater grazing time and reduced resting time in weed-infested paddocks compared to weed-free areas supports the notion that the animals changed their normal behavior and daily patterns of activities to adapt to the weed encroachment in the pasture. The same herbage allowance observed among treatments associated with the controlled effect of climatic factors on animal behavior, since all animals were under the same climatic conditions, demonstrate that the observed behavioral changes were an effect of the weed encroachment levels. In addition, the THI observed during the period of study (74–79) is considered to have had a light impact on heat stress for beef cattle [25], suggesting no effects of heat stress in our evaluations.

We estimated animals resting for, on average, 87.2% of the day, with daily grazing time and traveling time averaging approximately 7.6% and 5.2%, respectively. Although our estimates were calculated based on the speed ranges previously validated by Augustine [32] and adapted by Nyamuryekung’e [21], the data suggest that we have overestimated the resting time and underestimated the grazing time. This could be justified, because GPS loggers without tri-axis accelerometers cannot recognize smooth animal head movements in different directions (i.e., acceleration forces in axes x, y, and z) to accurately detect resting and grazing time. Thus, some grazing events were classified as resting when animals were grazing while standing at the grazing station or moving slowly (speed < 2.34 m/min) to another grazing station. Ref. [20] also used the same ranges of movement speed to classify cattle activity. They found an average resting time of 58% and grazing time of 34% during the summer, closer to the daily pattern of animal activity expected in grazing systems [22], likely because that study was conducted under similar conditions to the original calibrations. The traveling time estimated in our study (5.2%) is similar to that estimated by McIntosh [20]. Furthermore, it is within the range of time described by Kilgour [22] in a review of animal behavior in grazing systems (0.2 to 2.9 h). Indeed, GPS loggers without motion sensors (i.e., tri-axis accelerometers) are expected to be more accurate in terms of classifying traveling than resting or grazing activities. This is because animals cannot engage in two or more activities during periods of faster movements (speed > 25 m/min), whereas they can graze and rest simultaneously, possibly generating a confounding effect between resting and grazing classes. The combined use of GPS loggers and motion sensors can better detect immobility in grazing cattle [45].

Despite the possible inaccuracy of GPS collars in terms of estimating resting and grazing time, it is a crucial tool for monitoring changes in animal behavior in response to any challenge, such as weed encroachment in pastures. In weed-infested pastures, the focus should be on identifying changes in resting time and grazing time patterns according to the level of encroachment to detect the opportune moment for management interventions such as herbicide spraying or moving animals to another pasture area. In our view, this makes the device’s precision more important than its accuracy, since changes in the typical pattern of activity indicate the change in animal behavior regardless of the time spent in each activity. The GPS device showed applicability to monitoring animal behavior changes in response to pasture weed encroachment, but further studies are needed in order to calibrate a site-specific speed threshold to accurately estimate animals’ activities in grazing systems using low-cost devices without motion sensors.

### 4.3. Livestock Spatial Distribution

Although the estimated time spent by shady areas and water trough areas accounted for only approximately 33% of the day (Table 5), shady or water trough areas represent a small portion of the paddock area. The greater LPI in shady or water zones compared to bermudagrass or spiny pigweed areas demonstrates the preference of cattle for these areas when relating the daily time spent in each area to the proportion of the paddock area occupied by the respective zone of interest. Ref. [46] observed that grazing animals spent more time resting, grazing, and ruminating in areas near shade or water. Ref. [47] also demonstrated cattle’s preference for areas close to water, corroborating our findings. The ratio between time spent near shade or water and the respective ground area justifies the hotspots represented by the heatmaps in these areas. In addition, there were hotspots in areas close to the gates in the front corners of the paddocks, which generally have the lowest forage mass. This behavior is due to the hierarchy of behavioral drivers, with the lack of forage biomass in these zones offset by the animals’ curiosity about the gates [19,33].

The changes in the animals’ movement patterns throughout the evaluation periods in weed-infested and weed-strip paddocks suggest that the animals were avoiding weed patches and traveling mainly through the edges of the paddocks during the peak of the spiny pigweed development (periods 1 to 3), possibly because of the weed spines and the difficulty of walking and accessing the forage. This behavior was more evident with higher levels of weed encroachment (weed-infested paddocks). The greater time spent by animals in bermudagrass strips compared to spiny pigweed strips (Table 5) supports our statement that the animals avoided weed-infested areas. Some studies have reported animals avoiding spiny pigweed areas [12,13]. In addition, our field observations indicate that animals sometimes graze on the young leaves of the spiny pigweed, but quickly change grazing stations, possibly due to spines. Also, we observed that the bermudagrass growing beneath the spiny pigweed clusters was not grazed. 

Animals increased their spatial distribution over the paddock from the third period onwards in response to the spiny pigweed’s senescence. Spiny pigweed is an annual weed that usually reduces in biomass in mid-to-late summer, as was demonstrated in our study (Figure 4). The greater spatial distribution of the animals in the last evaluation period in both years, as well as the lower spiny pigweed HM in the last evaluation period, support this explanation. Corroborating our findings, Ref. [48] investigated the effects of weed control on animal distribution in a grazing system and reported cattle spatial evenness with a distribution up to five times greater in herbicide-treated pastures compared to non-treated pastures.

The more homogeneous spatial distribution of the animals during the five periods in weed-free paddocks compared to paddocks encroached upon by weeds, as observed in our study, demonstrates that pasture weed control can have additional benefits for the ecosystem. A greater spatial distribution of animals could play an important role in excreta distribution of livestock throughout the pasture, and could avoid excessive deposition of excreta in specific areas [49]. The decomposition of cattle excreta contributes to nutrient cycling in the soil, but excessive load may lead to soil degradation, nutrient leaching, and greenhouse gas emissions, in addition to compromising the forage growth [47]. This demonstrates the potential of GPS collars to track cattle distribution and guide grazing management for a more sustainable system. Rivero [19] suggested frequently repositioning water troughs, artificial shades, and supplement troughs in grazing systems as strategies to decrease the negative impacts of cattle distribution on soil conditions and greenhouse gas emissions.

This is the first study to evaluate the effects of pasture weed encroachment on animal activity and site preferences. Our findings have demonstrated the effect of weed encroachment on animal behavior and the importance of weed control in grazing systems. GPS tracking collars could possibly detect changes in livestock spatial distribution and daily activities according to the level of weed encroachment in the pasture, allowing for the detection of potential challenges to animal welfare in grazing systems and supporting decision making for management interventions, such as weed control. We emphasize that the GPS collars were used to monitor the effect of weeds on animal behavior on a high temporal scale, without human interference in animal activities. However, we did not evaluate the long-term effect of increasing animal activity on energy balance. Further effort should be devoted to evaluating the economic viability of GPS collars for cattle tracking, determining the most suitable number of tracked animals for herd monitoring, and estimating the impact of energy expenditure on increasing activity in long-term animal performance.

## 5. Conclusions

Pasture weed encroachment affects animal behavior and spatial distribution in grazing systems. Animals in weed-infested pastures tend to reduce their resting time and increase their grazing time, distance traveled, and rate of travel as a possible adaptation to weed encroachment. GPS tracking collars are alternative tools used to monitor animal behavior grazing systems in order to provide near real-time information to support decision making for management interventions. Further long-term studies are encouraged to evaluate the impacts of weed encroachment on animal performance, quantify the impacts of behavioral changes on animal energy balance, and determine the economic threshold for weed control in grazing systems.

## Figures and Tables

**Table 1:** The starting date of each evaluation period during two cycles of monitoring (summer 2021/2022).

Evaluation Period	Date	
	Cycle 1	Cycle 2
1	6 July 2021	12 July 2022
2	28 July 2021	3 August 2022
3	19 August 2021	25 August 2022
4	10 September 2021	16 September 2022
5	-	8 October 2022

**Table 2:** Forage and animal responses during the study period (summer 2021 and 2022) in different levels of pasture weed encroachment.

	Weed-Free	Weed-Strip	Weed-Infested	SEM	*p*-Value
Bermudagrass HM (kg DM ha^−1^)	2993	3114	3021	98	0.50
Spiny pigweed HM (kg DM ha^−1^)	-	1024 b	2740 a	383	<0.01
Total HM (kg DM ha^−1^)	2993 c	4138 b	5761 a	644	<0.01
ADG (kg hd^−1^ d^−1^)	0.28	0.36	0.26	0.05	0.44
Stocking rate (AU * ha^1^)	4.7	4.7	4.5	0.12	0.39
Herbage allowance (Kg DM kg^−1^ BW)	2.1	2.2	2.2	0.10	0.08

LSMeans followed by different letters in a row are significantly different (*p* < 0.05). Abbreviations: HM (herbage mass); ADG (average daily gain). * AU (animal unit): 1 AU = 350 kg BW.

**Table 3:** Daily average distance traveled and rate of travel of animals in paddocks with different levels of weed encroachment.

Item	Weed-Infested	Weed-Strip	Weed-Free	SEM	*p*-Value
Distance traveled (m/day)	5657 a	5079 ab	4482 b	417	0.02
Rate of travel (m/min)	6.0 a	5.0 b	5.1 b	0.60	0.045

Means followed by different letters in a row are significantly different (*p* < 0.05). Abbreviation: SEM (standard error of the mean).

**Table 4:** Percentage of daily time spent by animals in each activity in a grazing system with different levels of weed encroachment.

Item	Weed-Infested	Weed-Strip	Weed-Free	SEM	*p*-Value
Resting time (%)	85.1 b	87.8 ab	88.8 a	1.36	0.03
Grazing time (%)	8.9 a	7.2 b	6.6 b	0.86	0.02
Traveling time (%)	6.0	4.9	4.7	0.63	0.09

LSMeans followed by different letters in a row are significantly different (*p* < 0.05). Abbreviation: SEM (Standard error of the mean).

**Table 5:** Percentage of the day spent in shady areas, water trough areas, and in different strips of the paddocks by animals in a grazing system with different levels of weed encroachment. Estimates of time spent in each area are averaged across five evaluation periods and two years of study for each treatment. Landscape preference indexes (LPI) are descriptively presented by averaging treatments.

Time Spent (%)	Weed-Infested	Weed-Strip	Weed-Free	SEM	*p*-Value	LPI
Shade	23.4	20.0	20.2	3.4	0.7	13.8
Water	6.8	13.5	12.6	2.6	0.2	28.5
Bermudagrass strips	-	37.3 a	-	4.6	0.01	0.7
Spiny pigweed strips	-	29.1 b	-			0.6

LSMeans followed by different letters in a column are significantly different (*p* < 0.05). Abbreviations: SEM, standard error of the mean; LPI, landscape preference index.

## Data Availability

The data that support the findings of this study are available from the corresponding author upon reasonable request.
